# The Free2B Multi-Media Bullying Prevention Experience: An Exemplar of Scientific Edutainment

**DOI:** 10.3389/fpsyt.2020.00679

**Published:** 2020-07-16

**Authors:** Stephen S. Leff, Tracy Evian Waasdorp, Brooke S. Paskewich, Katherine B. Bevans, Flaura K. Winston

**Affiliations:** ^1^The Violence Prevention Initiative, Children’s Hospital of Philadelphia, Philadelphia, PA, United States; ^2^The Perelman School of Medicine, The University of Pennsylvania, Philadelphia, PA, United States; ^3^The College of Public Health, Temple University, Philadelphia, PA, United States

**Keywords:** scientific edutainment, edutainment, bullying, prevention, school-based, community-based participatory research

## Abstract

**Objective:**

The objective of the current article is to highlight an example of a new paradigm, *Scientific Edutainment*. The manuscript describes how educational researchers and technologists worked together to develop a multi-media bullying prevention experience, called Free2B for middle school students paying particular attention to ensure that the programming was not only relevant to all students but also was appealing and responsive to the needs of urban youth. Bullying is the most common form of aggression experienced among school-aged youth, which impairs students’ learning and social-emotional functioning and has financial costs to society. Given that the prevalence of bullying is highest in middle school, finding brief and feasible methods for motivating and sustaining change at this age is critically important, especially in the case of urban, under-resourced schools.

**Method:**

In response to this challenge, multidisciplinary bullying prevention researchers collaborated with international technologists to develop the Free2B multi-media bullying prevention experience through an iterative Community-Based Participatory Research (CBPR) approach. In addition, the research team conducted a series of pilot studies to iteratively develop and initially evaluate the multi-media program, helping to ensure relevance specifically for urban middle school youth.

**Results:**

Results from the pilot studies indicated that the vast majority of middle school students found the Free2B multi-media bullying prevention experience to be enjoyable, relevant to their needs, and addressed important strategies to handle peer bullying and victimization. In addition, the brief prevention experience was associated with increases in problem-solving knowledge, prosocial attitudes about bullying, increased sympathy, and confidence in handling peer conflicts.

**Conclusion:**

The current paper illustrates the use of a new paradigm, termed *Scientific Edutainment*, as a way to combine evidenced-based developmental science with the latest in entertainment technology to provide innovative, engaging, and technologically-sophisticated educational programming.

## Introduction

The term Edutainment has been used relatively frequently over the past 15-20 years to broadly describe the combination of education and entertainment in order to help children and adolescents, and sometimes institutions and/or other entities, learn and promote new skills (1–6). Some have emphasized that Edutainment denotes that the learner is actively engaged in their learning through the entertainment and technology aspects (7), and that the teaching of new skills can occur in any setting and not just within a classroom or school context (7). Thus, there has been an emphasis on the use of interactive and immersive technologies (e.g., augmented reality, immersive virtual reality, mixed reality environments) with the idea that the entertainment technology can stimulate stronger cognitive engagement from participants thereby helping to facilitate the learning process (8, 9). These interventions have been applied with some level of success to a range of different topics, including the promotion of safer teen driving (4), health education related to HIV and AIDS in 3^rd^ world countries (10), sexual abuse prevention (11), and preschoolers’ reading skills (12). The strengths of Edutainment approaches are that they may enhance creativity, transform traditional learning into interactive and immersive learning experiences, improve participant engagement and motivation, and utilize the latest innovations in technology. However, from our perspective, the strengths of Edutainment can be negated if best practice science and strong theory are not used to design the content delivered. In those cases the entertainment value may be high but the effectiveness and generalizability of the intervention strategies may be limited. As such, we refer to our work throughout the remainder of this paper as “*Scientific Edutainment*” to indicate that this signifies best practice science and theory combined with strong educational practices and the latest in entertainment technology.

The goal of the current manuscript is to provide an example of *Scientific Edutainment*, in which bullying prevention researchers partnered with an international technology team to develop a multi-media bullying prevention experience (called Free2B) for middle school students, given that bullying peaks during these years (13, 14), paying particular attention through a community-based participatory research (CBPR) process to ensure that the program not only had global relevance but also was engaging and responsive for urban youth and educators. Descriptions for how program materials were developed and initially evaluated will be described through a series of pilot studies. Through this process we will highlight how researchers can collaborate with multi-media experts to develop and iteratively evaluate and adapt materials to ensure that resulting programs are both engaging and scientifically-grounded. Challenges for researchers in trying to bridge the gap between theory, practice, and innovation will be highlighted, including the need for balancing the use of technology to engage students in learning best practice strategies in an entertaining way without depicting extreme violence.

### The Problem of Peer Bullying

Peer bullying at school is a significant childhood experience that is associated with considerable behavioral, social, and academic difficulties that can be prevented and/or ameliorated through systematic prevention and intervention programs (15, 16). Bullying prevention programming for middle school students is particularly important given that bullying increases in late childhood and peaks in early adolescence (13, 14). This makes the middle school years an extremely important time period to intervene to help suppress this increase and lessen the impact of bullying. This is especially true for urban minority youth, as previous research suggests that programming has not typically been adapted to be culturally-responsive to the needs of many of these high-risk youth (15, 17, 18). A critique of empirically-supported bullying prevention programs is that some educators feel that programs are too time- and labor-intensive, not engaging to students, as well as not being culturally-relevant for urban minority youth (17, 18).

Brief technology-based bullying prevention experiences may play a role in building youth knowledge of bullying prevention facts while promoting students’ attitudes that they can play an important role in reducing bullying behaviors at their school. These approaches can simultaneously provide survey results so that they each school can have a data-informed approach to addressing their school’s unique bullying climate and culture, and illuminate next steps needed to create more lasting change during adolescence. As such, we developed and pilot tested an empirically-supported multi-media bullying prevention experience to support middle school students in the initial stages of bullying prevention programming through initiating collective action and evidence-based decision making. This paper details how a *Scientific Edutainment* experience was developed through CBPR as researchers and technologists partnered in the iterative design of this prevention experience. A CBPR approach combines psychological theory and best practice science with key stakeholder feedback (17), and it is similar to prior research focused on engaging youth in collaborative decision-making techniques to determine intervention preferences (19, 20). Results will be presented from a series of pilot studies that were used to ensure that Free2B is universally relevant yet scientifically rigorous and sensitive to minority youth living in urban communities.

### Strengths and Weaknesses of Published Research in Bullying Prevention

Although all states have mandates requiring schools to address bullying (21), there is great variability in how schools accomplish this. Common approaches are to conduct stand-alone anti-bullying programs or a single-session assembly (22). Stand-alone bullying programs, have historically been developed and conducted in European countries (e.g., Olweus’ Bullying Prevention Program) but have become more common in the United States over the past 15 years (16, 23). Strengths of some of the most well-known bullying prevention programs are that they are theoretically grounded and they include the necessary elements for bullying prevention (24–26). For instance, best practice programs address broad school climate, aim to improve supervision and monitoring in the unstructured school settings, support clear and consistent rules preventing bullying, and involve all students, school staff, and parents in supporting these efforts. Despite this, many of these efforts have resulted in relatively small reductions of bullying (16, 25) with effects declining in older adolescents (27). Limitations of stand-alone programs, even the more successful ones, are that they are labor-intensive to implement fully or as intended, especially outside of the context of a well-controlled research study, sometimes resulting in suboptimal impact (16, 28). For these reasons, use of a well-respected bullying prevention program often does not translate into positive changes. This is particularly true for urban under-resourced schools that grapple with additional stressors, such as single-parent homes, poverty, and community violence (17).

As an alternative, schools will turn to quick fixes such as a school assembly. Assemblies appeal to schools because of their minimal time commitment and lack of burden on busy teachers. However, assemblies have several inherent limitations. First, many existing bullying prevention assemblies are “lecture-style” and therefore are variably engaging. Further, many use punitive messages and reprimand bullying behavior (e.g., a zero tolerance approach), which is a reactive response to bullying (22, 29) that does not engage students to create a lasting impact when used alone (23, 29). Assemblies are often not theoretically-grounded and it is not always clear how they have integrated best practice bullying prevention core content of problem-solving, perspective-taking, sympathy, and instructions for bystanders of bullying (16, 23). This makes it impossible for assemblies to be systematized and/or scaled. Finally, an assembly is rarely coupled with data-collection that could be used to provide tailored feedback. In sum, while convenient, assemblies often lack the theoretical foundation and a positive, engaging approach that is necessary to capture student interest and foster behavior change.

>In summary, the goal of this paper was to describe how researchers and technologists can work together to establish engaging programming that is theoretically-based and empirically supported. In order to accomplish this goal, we describe how brief bullying prevention programming was developed through an iterative partnership-based approach to ensure that the result would be scientifically-grounded, theoretically-based, and make use of innovative technology to engage students in a 90 min interactive learning assembly about bullying prevention programming.

## Methods and Iterative Development of Programming

All aspects of the project described in this manuscript were approved by the authors’ institutional review board (IRB). As such, all students at participating schools received the multi-media programming and completed pre- and post-questions anonymously on a hand-held remote device. Children who participated in focus groups were required to obtain parental permission (and child consent) prior to participation.

### Initial Partnership

Researchers with considerable expertise in intervention development for aggression and bullying prevention programming were approached by an international technology team who had extensive experience in developing interactive educational programming for youth. The technology team approached the researchers with their desire to develop a brief multi-media bullying prevention experience for middle school-aged youth, given the high prevalence of bullying during these years (13, 14). Each partner (e.g., the research team and the technology team) brought particular expertise and limitations to the collaboration. For example, the research team had experience in program design and methodology, psychological theory related to intervention programming, and knowledge of empirically-based best practice strategies for bullying prevention programming. In addition, the research team had substantial experience working in urban school environments, developing effective evidence-based universal and indicated aggression and bullying prevention programs (17, 30, 31) and had a good understanding of strategies for anti-bullying programming that are considered ineffective and/or could “cause harm” by scaring students as opposed to engaging and/or teaching them (32, 33). However, the research team had limited experience working with technologists and producers and were not familiar with the production process and related time-lines.

The technology team had notable strengths in developing 3D interactional experiences related to educational topics, knowing the latest in technological advances, and having considerable experience developing, producing, and scaling programming through a portable school-based assembly-style format. The technologists had also worked with research teams in the development of their prior programs, which allowed the current research team to build upon this foundation in developing a systematic and iterative process for the development of Free2B using the community-based participatory research (CBPR) model. Given that a CBPR approach can lead to stronger and more culturally-sensitive programs, but invariably also be a slower process, the research team had to figure out how to provide meaningful data-driven advice quickly and efficiently so that the production portion of the team could meet projected timeline goals. Many times this was accomplished by having the research team prioritize feedback given to the broader team in order to emphasize which aspects were most crucial.

In sum, the initial partnership took a number of meetings across several months whereby leaders of both teams met together to speak openly about the ways in which they liked to work, their respective strengths, and projected challenges. The end result was the agreement to have weekly virtual “working meetings” between lead researchers and technologists to further establish goals, timelines, and ways to communicate and collaborate most successfully.

### Developing Working Relationship and Goals

Early weekly meetings included discussing how a brief multi-media prevention program could be used to increase student awareness of bullying and motivate students to be ready for making changes to their school climate. The research team emphasized the importance of taking a positive-based approach to the project which would likely foster more engagement and change (34, 35) rather than a fear-based approach trying to scare children (29) that has often been used in docudramas and popular media. For example, researchers suggested highlighting the positive implications and power that students could gain back from a child who bullies by being a positive and proactive bystander as opposed to highlighting the negative effects of depression and suicide that peer victims can experience. Over the course of several months, A Memorandum of Understanding (MOU) was developed between the research and technology team that outlined the goals, expectations, and proposed production schedules. For instance, the MOU indicated that the research team would be responsible for developing a white paper and logic model that would help articulate the main concepts and constructs to be illustrated in the multi-media production (see below for a more detailed description), and that the white paper and logic model would be used to help ensure that different aspects of the programming were grounded in the empirical literature on best-practice strategies for peer bullying prevention. These same concepts and constructs were used to help determine outcome metrics. At the same time the MOU laid out production time-tables and the detailed type of feedback the technologists required from the research team for iteratively developing components of the program (e.g., including drafting of scripts, story-boarding, focus group feedback, and production schedules).

### Generation of a White Paper on Bullying Prevention

**Figure 1:**
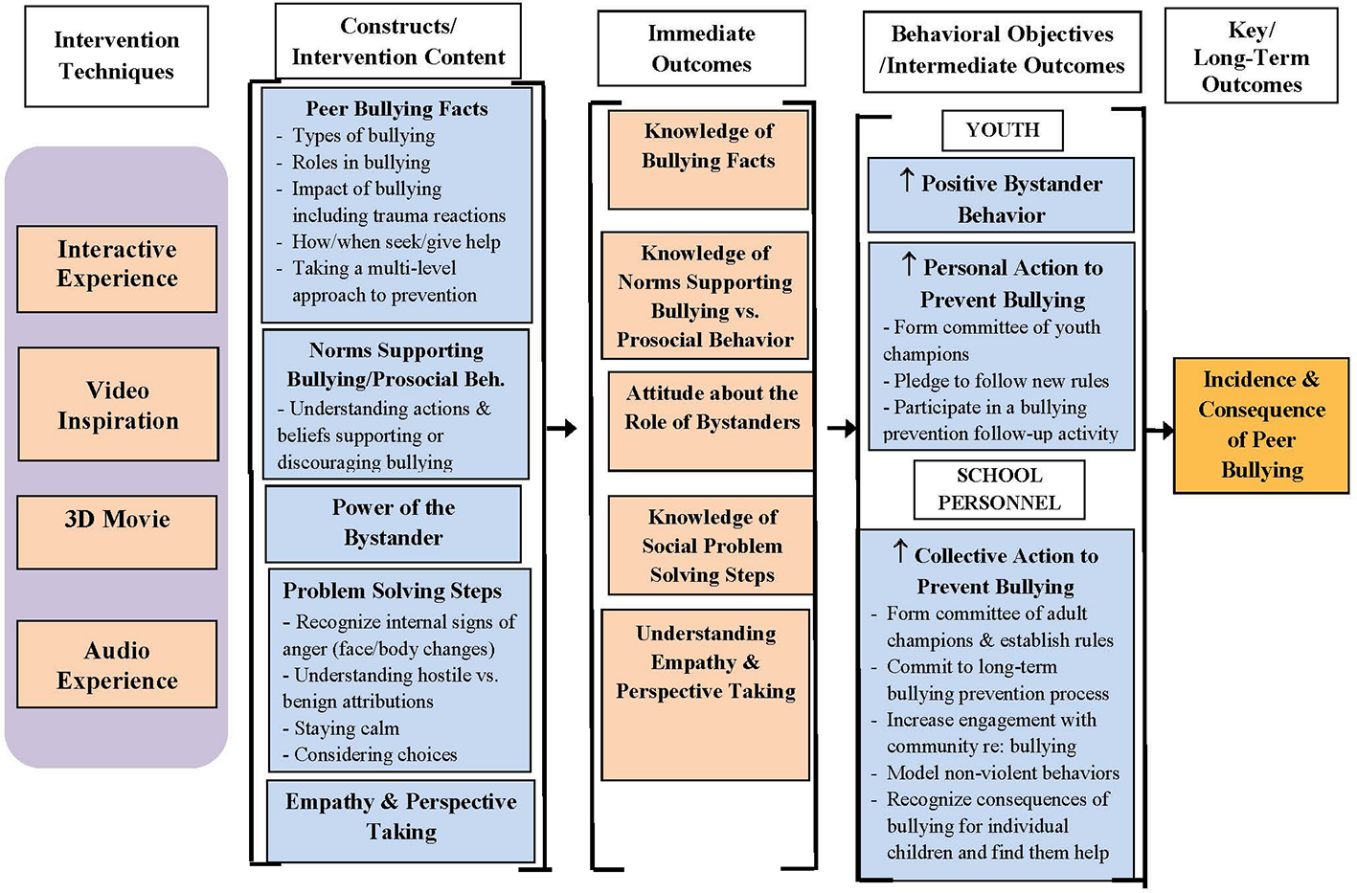
Program theory for Free2B: Decreasing incidence and consequence of peer bullying.

The research team then worked for several months to develop a White Paper (e.g., Concept Paper) to clearly articulate the scientific foundation for the multi-media program in bullying. This document included: a) key background literature review and summaries related to bullying and victimization; b) diagrams and articulation of the program theory (see **Figure 1**); c) details on recommended content and associated constructs based upon best practice scientific principles related to peer bullying prevention programming; d) projected immediate, intermediate, and long-term behavioral outcomes for the program^1^; and e) representative items to utilize as part of a pre- and post-test interactive survey.

### Description of Prevention Experience Components

The goal for the team working together to design the Free2B bullying prevention experience was to develop a 90 min multi-media bullying prevention experience grounded in best practice science and relevant psychological theory. In general, meetings occurred about once per month over approximately 6 months with specific tasks laid out for the research team and for the technology team between meetings. The original prevention experience that was rolled out in pilot study #1 described below consisted of four primary intervention components and 13 pre- and post-assembly questions which were completed using the interactive hand-held devices. First, an engaging 3D movie that highlights the harmful impact of all forms and modes of bullying (e.g., physical, relational, and cyber) and the role that positive bystanders can play in helping to promote a safe school climate. This included the “director’s cut” following the 3D movie, where the director, actors and actresses talk openly about the impact that peer bullying has had on their lives, how it directly affected many of them growing up, and the steps they took to overcome and/or try to make it better. Second, video testimonials in which adolescents share their bullying and victimization experiences through social media (e.g., simulating a YouTube^©^ posted video displaying thoughts on index cards) in an effort to inspire students’ to take a stand against bullying. Third, an interactive quiz show in which youth learn basic knowledge about bullying, emotion regulation, and being a positive bystander. During the quiz show the youth answer multiple choice questions using interactive hand-held devices, questions include concepts related to myths and facts of bullying, how to recognize when they are becoming angry, how to best evaluate social situations before reacting quickly and/or impulsively towards others, and strategies for being a positive and proactive bystander. Finally, the dark room audio experience, during which time students hear a story in a darkened room so that they must use their auditory senses to listen, learn, and react to a story as it unfolds.

All participants completed a 13-item pre- and post-program questionnaire (outside of the interactive quiz show) through the interactive hand-held remote devices, viewed the 3D movie, listened to the dark room audio experience, and engaged and actively participated in the interactive experiences.

### Underlying Program Theory of the Prevention Experience

Three psychological theories, social information processing (36), developmental-ecological (37, 38), and cognitive-behavioral theories (39, 40), as described below, were combined with a positive approach to bullying prevention in order to provide a theoretically-grounded and engaging learning experience for students. Social information processing (SIP) models of aggression suggest that a child approaches each interpersonal situation through a combination of biologically determined capabilities, memories of prior practices, and models for social situations. A child’s behavioral response in a particular peer interaction is posited to be a function of how these pre-existing capabilities interact with the way in which children process a series of social cognitive steps (36). Free2B is grounded in an SIP re-training framework, modeled after attributional re-training programs, such as the Coping Power Program (41), the Brain Power Program (42), and Friend to Friend (17, 30). These programs were chosen because they have shown aggression reduction among urban African American youth. For instance, the interactive quiz show component of Free2B focused upon illustrating several basic social problem-solving strategies including how to recognize when you are getting angry, how to slow yourself down and examine social situations prior to acting, and how to give others the benefit of the doubt when their motives are unclear. Bronfenbrenner’s developmental ecological theory (37, 38) and more recently the bioecological theory (43, 44) also influenced the design of Free2B. This model suggests that development is influenced by relationships and interactions with significant others in one’s social environment. As such, Free2B was designed to motivate and change the behaviors of the bystanders of bullying (both youth and adult) such that these individuals interact more positively when confronted with bullying. Finally, cognitive behavioral strategies (observing behaviors through the 3D movie and inspirational videos; shaping new behaviors) derived from social learning theory (39, 40) were also used to make Free2B engaging and impactful. For example, the video testimonial and the “director’s cut” components (components 1 and 2 listed above) of Free2B were designed with the idea that by observing other youth and young adults successfully handling and/or talking about how they handle bullying would provide a model for how the students themselves could use positive bystander techniques to enhance school climate.

Researchers developed the program theory (see **Figure 1**) and primary teaching content, consulted with a local youth advisory group, and developed evaluation procedures^2^. They also recommended that the program target middle school youth, as this is the time in which rates of bullying are the highest (13, 14). The program theory illustrates how all four primary intervention components are thought to impact both proximal (e.g., knowledge of bullying facts; prosocial attitudes about positive bystander behavior) and distal (e.g., increases in positive bystander behavior and collective action to prevent bullying) outcomes. As program materials were being developed the technology team asked for more detailed guidance as to the main teaching points the researchers hoped to achieve within the program. As a result, the researchers developed a Most Important Concepts/Key Teaching Points document (see **Table 1**). This helped to articulate the main constructs and take-away messages that needed to be covered in one or multiple components of the intervention in order to ensure that the content was covered and emphasized in a scientifically-grounded manner.

**Table 1:** Most important concepts and key teaching points.

Key Point	Description	Broader Construct	Take Away Message
#1	Defining Bullying	Knowledge/Myths re: bullying	-Bullying is aggressive behavior that occurs repeatedly in context of a power imbalance
#2	Subtypes of Bullying	Knowledge/Myths re: bullying	-Physical (hitting, kicking, threatening), Relational (harming others by damaging reputation through gossip, social exclusion) -Verbal (insulting through words) -Cyber (Using technology to harm others)
#3	Bullying hotspots at school	Knowledge/Myths re: bullying	-Occurs most often in unstructured settings (e.g., lunchroom, hallways) when adults are not present
#4	Who is a Bully or Victim?	Knowledge/Myths re: bullying	-Anyone can be a bully or victim (can’t tell by how someone looks), and bullies are often quite popular & socially influential despite not being well-liked
#5	Impact of bullying	Norms supporting prosocial as opposed to bullying climate	-Bullying has a negative impact on behavior, class climate, academics, & social relations
#6	Preventing bullying & improving school climate	Norms supporting prosocial as opposed to bullying climate	-Necessary for youth, diverse school personnel, & parents to work together to develop positive ways of encouraging peer interactions, establishing clear rules to prevent bullying, & forums for discussing concerns
#7	Bullying is about Power	Understanding the unique role of the Bystander	-Bullies have the power, victims have little power, and bystanders don’t realize their power potential (e.g., bystanders can have power by exhibiting prosocial behaviors and messages)
#8	Teaching a series of problem-solving steps	Knowledge of Problem-Solving	-Recognizing our own body language when becoming angry/upset -Staying calm (e.g, taking deep breaths, using visual imagery, counting to 10) -Looking at each situation closely (not just assuming others “meant” to be mean or aggressive) -Considering our choices in social conflict situations
#9	Seeing others’ points of view	Perspective-Taking	-Important to consider other’s perspectives
#10	Recognizing others’ feelings	Empathy	-Recognizing that behavior impacts others’ feelings

## Iterative Pilot Studies Results

The first pilot study of Free2B was conducted at two urban middle schools serving ethnic minority students (121 8^th^ graders) within a large urban school district. All components of the intervention had been fully developed through the partnership previously described, and although quantitative data was collected and evaluated as part of this initial implementation, the focus was on ensuring the acceptability, relevance, and feasibility of the 90 min program. Further, the goal of this pilot was to obtain qualitative feedback from randomly selected 8^th^ graders, teachers, and counselors from each school who participated in focus groups immediately following the multi-media experience. Both quantitative data and focus groups with students and staff indicated that Free2B was engaging and enjoyable, and that the show enhanced students’ knowledge and prosocial attitudes about bullying. Despite this, students reported that they could not fully relate to some of the characters and/or settings in the show. This was important feedback, for which a CBPR approach was used to ensure relatability and relevance with urban minority youth by making slight adaptations to Free2B including: 1) re-filming the video testimonial component with a more diverse group of actors and actresses, 2) depicting more contextually relevant themes for bullying that any student audience should be able to relate to (e.g., the original video testimonial had the youth being bullied for having red hair, this was changed to being bullied for being overweight and not having nice clothes in the revised version), 3) enhancing the visibility and roles of the minority characters in the 3D component, and 4) adapting several visual prompts on the interactive quiz show in order to better highlight the main teaching points and constructs. Through the qualitative feedback, overwhelmingly students did not find the dark room component as informative or engaging as compared to the other components. For instance, they found the story hard to follow, having trouble differentiating the different voices and characters and at times finding the story too complicated. As a result, the research team suggested that the revised program not include the dark room experience, or that this component be substantially revised and adapted.

Following the iterative changes described above, a second pilot study was conducted with 714 7^th^ and 8^th^ graders from five middle schools. These schools were chosen in order to ensure that there was diversity in school type (urban versus suburban) and in terms of school neighborhood (e.g., SES level, rates of violence in community). Of the five schools, two were urban low-income schools, one was a suburban school with a moderately high SES, and two were suburban schools in relatively impoverished neighborhoods. All aspects of the 90 min experience were conducted and results indicated that 88% of students found Free2B to be enjoyable, 92% thought it taught helpful strategies to stop bullying, and 85% indicated that it addressed issues important to them. Significant paired sample t-tests and McNemar χ2 also suggested that Free2B produced immediate post-assembly changes related to increased social problem-solving knowledge, prosocial attitudes about bullying, increased sympathy, and confidence in resolving conflicts (see **Table 2**). Further, focus groups with participating students in the two urban schools suggested that changes to Free2B after the 1^st^ study, made it more culturally-relevant, relatable, and impactful. For example, students reported that they were able to relate to characters depicted in the 3D and video testimonial parts of the program in line with the changes made following pilot study 1.

**Table 2:** Pre and post Free2b experience scores on selected items.

Likert Questions^a^	Pre Mean	Post Mean	Paired *t*	*p* –value
It is my responsibility to help students who are bullied.	2.30	2.42	-3.30	<.001
I could help someone who was bullied.	2.84	2.94	-2.88	.004
How bad would you feel for a student who was bullied?	2.91	3.07	-5.09	<.001
**Dichotomous Questions**	**Pre % Correct**	**Post % Correct**	**McNemar χ2**	***p***
Bullying is a normal pat of growing up (correct answer: False)	40%	72.1%	165.42	<.001
What is the BEST way to keep calm in an argument? (correct answer: Take deep breaths)	27.8%	52.5%	96.46	<.001
When you’re having an argument, what is the BEST reason to pay attention to other student’s face and body? (correct answer: Because it can help you figure out how he/she is feeling)	46.6%	63.4%	62.11	<.001

Note. ^a^Items were on a scale from 1 = not at all to 4 = a whole lot.

A 3rd pilot study was conducted with 1155 6th grade students from eight middle schools in a large predominately minority urban school district in another part of the country. Results produced similar positive results to those described in pilot study 2 above. For instance, 87% of students found Free2B to be enjoyable, 93% thought it taught helpful strategies to stop bullying, and 87% indicated that it addressed issues important to them. Finally, significant paired sample t-tests and McNemar χ2 were found across the same domains outlined above. The similar positive results obtained demonstrated that the program has promise with younger (6^th^ graders) predominately urban youth and across different geographic regions of the country.

## Discussion

The primary goal of the project was to iteratively develop and preliminarily evaluate an engaging, interactive, easily administered experience aimed at bullying prevention that is effective for all youth, with particular focus on the relevance for an urban minority population given that programs are not always responsive to these children’s needs and concerns (17, 18). In order to accomplish this a *Scientific Edutainment* approach was utilized which combined state-of-the-art entertainment technology, a strong theoretical foundation applied to bullying prevention research, and CBPR with youth and educators. The program was continually adapted and fine-tuned through stakeholder feedback in order to ensure engagement, relatability, and relevance for urban ethnic minority youth in addition to other student audiences.

The current manuscript highlights a number of advantages as well as challenges for using a *Scientific Edutainment* approach to program development. Strengths of this approach include that it is a paradigm that allows for the integration of multiple disciplines and fields to work together to ensure scientific rigor as well as strong youth engagement and entertainment value. Further, this paradigm illustrates how this approach can be used to address a gap in the field of bullying prevention for middle schoolers; that is, how to utilize the assembly-style format which is feasible and brief (that schools continue to use despite the availability of evidence-based effective programming) in a way that is systematic, theoretically-grounded, data-driven, and designed to provide clear teaching and training strategies for bullying prevention without glorifying violence or inducing fear in youth. The integration of technology and entertainment into the program also ensures that students are provided materials and teaching concepts through modalities and techniques of which they are familiar. This study also provides a model for how educational researchers and technologists representing the entertainment field can work together through a CBPR format to impact youth positively while ensuring that they are “doing no harm.”

An additional strength of this *Scientific Edutainment* approach is that a multi-media experience such as the one presented here can be fully implemented with strong fidelity across diverse school types and settings as it only requires a large auditorium or gymnasium. Given that the 90 min experience simulates a film or movie, it requires only an MC to help redirect students and/or to answer questions if needed as the program begins. This helps to ensure that all aspects of the experience occur during each program showing. As such, the systematic procedures and high treatment fidelity which is built in to this system also addresses inherent limitations for typical school assembly programs (e.g., variable content and presentation styles, different presenters) as well as more established stand-alone programs which are often infeasible to implement as intended outside the context of a large research grant or trial (16).

There were a number of challenges that also are illustrated through the current use of a *Scientific Edutainment* approach in the current study. First, utilizing the most engaging technology while keeping the budget to a reasonable scope was a challenge, especially when utilizing a CBPR iterative approach to project development. As a result, the combined research and technology team agreed that they would provide suggestions based on their past experiences and/or quantitative or qualitative data by organizing concerns into different domains for prioritization. These included feedback and changes that were: 1) absolutely essential because if not changed they may send the wrong message and/or cause harm; 2) essential for more clearly articulating valuable teaching content or strategies; 3) non-essential but suggested in order to potentially strengthen program effects; and 4) non-essential but if budget allowed would make the final production more systematic or professional but would likely have no major impact on program effects. Given the strong relationship between the teams, researchers and technologists were able to work closely together to avoid any priority #1 issues by articulating that the goal was to motivate students to want to make a change at their school, and therefore all team members agreed that it would be much more important to tell stories of hope, of overcoming obstacles, and teaching of feasible strategies as opposed to showing the worst case scenarios for bullying victims (e.g., depression and suicide; homelessness). Given these early conversations through the CBPR process there were no times where researchers or technologists were fearful that the program could cause harm. In contrast, there were a number of times in the early stages of development where researchers used iterative data or qualitative feedback to suggest changes to the way teaching points were phrased or presented on screen, and to the diversity and/or main messages of the characters, in order to help ensure that the main teaching content was optimally portrayed and presented to diverse audiences.

It should be noted that there was a steep learning curve for both groups due to the unfamiliarity as to the production process and time-table for researchers, and to the ways in which CBPR research teams work to represent youth voice and input at each stage of the iterative development process for the technology and producer team. Weekly virtual meetings, frequent discussions, and sharing of documents (e.g., scripts, white paper, examples) helped bridge this gap in experience for both teams. By virtue of having a close partnership between teams, the initial feedback obtained from urban students in pilot study 1, which necessitated making some changes to aspects of the program components (e.g., re-filming the video testimonial component with a new set of actors; emphasizing to a greater degree the important role held by the minority actors within the 3D film), was able to be utilized in a quick and efficient manner prior to pilot study 2.

The current study served as an illustration of the *Scientific Edutainment* paradigm. This paradigm has direct implications for bullying prevention programming and related interventions. While we recognize that any single-day experience is unlikely to reduce bullying alone^3^, it is clear from these pilot studies that Free2B is unique in its ability to produce very strong immediate changes that require minimal reinforcement or involvement from teachers or school personnel. However, as suggested by best practice science and our focus group feedback, programming should go beyond a 1-day assembly in order for schools to promote and maintain an anti-bullying climate. Given that Free2B utilizes handheld devices to collect student self-reported data pre-, post- and during the interactive quiz show portion of the program, this information can be utilized to help schools move beyond a 1-day program. For instance, in the future, this data could be used to customize “school bullying need reports” based upon each school’s data. The use of this data will allow for near real-time comparative studies of bullying surveillance and advance our understanding of bullying in a wide range of contexts. Further, in an age of data-based decision making in the schools the use of school-specific data to personalize planning and action steps is a crucial component that is missing from most current “one size fits all” bullying prevention programs. Finally, the use of school specific data could be used to determine current prevention programming success, plan for future programming, and/or track progress over time. Thus, future research examining the Free2B experience when combined with “school bullying needs assessment reports” can be helpful for planning and choosing more intensive or targeted bullying prevention efforts that would be important in capitalizing upon the positive initial steps from Free2B and promoting longer-term behavioral change in the schools.

Based upon our experience in using a *Scientific Edutainment* paradigm to develop bullying prevention programming we have several recommendations for helping educators think through their use of assembly-style programs to address peer bullying including that it: a) draws upon a strong scientific foundation, b) uses a positive and interactive approach which may help facilitate learning as opposed to a “fear-based” approach, and c) provides data back to schools that could include online and free resources for students, school staff, and parents. In addition, it is recommended that school personnel come up with a list of discussion questions to help students better articulate and apply the concepts learned, and use follow-up surveys to ascertain student feedback and understanding.

In summary, Free2B demonstrates rigor in its series of pilot studies, use of CBPR to ensure relevance and meaning to urban youth, and significant findings across samples varying in grade and geographic location. The use of the data collected during Free2B may address the limitation of the brief program by expanding beyond the focus on immediate changes and providing schools with a data-driven approach to influence the bullying climate through collective action and positive bystander behavior. The *Scientific Edutainment* approach utilized in the current research holds promise for detailing how diverse groups of educators, researchers, and entertainment industry groups can work together to design innovative, scientifically-grounded, and engaging means for addressing key educational problems such as peer bullying through future research.

## Data Availability Statement

The datasets generated for this study are available on request to the corresponding author.

## Ethics Statement

The studies involving human participants were reviewed and approved by The Institutional Review Board, Children’s Hospital of Philadelphia. Written informed consent to participate in this study was provided by the participants’ legal guardian/next of kin.

## Author Contributions

SL, TW, BP, KB, and FW conceptualized the study and prepared the manuscript. BP and SL recruited schools for participation and oversaw study implementation, and TW conducted data analyses. SL and TW played a key role in writing the paper with critical review provided by FW. SL, TW, BP, KB, and FW gave final approval for the submitted version.

## Funding

This research was supported in part by an NIH grant, 1R01HD094833-01A1 and by the Children’s Hospital of Philadelphia. This research was made possible, in part, by the support of the School District of Philadelphia in whose schools data collection and showings of the Free2B experience occurred. Opinions contained in this report reflect those of the authors and do not necessarily reflect those of NIH, Children’s Hospital of Philadelphia, Life Changing Experiences, and/or the School District of Philadelphia.

## Conflict of Interest

The authors declare that the research was conducted in the absence of any commercial or financial relationships that could be construed as a potential conflict of interest.

## Footnotes

1 Intermediate and long-term outcomes were outlined with the expectation that the multi-media experience could be combined with more intensive ongoing programming to make lasting behavioral change.

2 All items used for pre- and post-testing were selected from validated measurement tools by examining their psychometric properties in past studies combined with the cognitive testing of the specific items with small numbers of middle school youth to ensure adequate understanding.

3 Free2B was not examined for its potential long-term impact and/or influence on bullying behaviors themselves, which could be a goal for future research.
